# Phytochemical investigation and anti-inflammatory potential of *Atriplex leucoclada* Boiss

**DOI:** 10.1186/s12906-023-04281-5

**Published:** 2023-12-16

**Authors:** Hayam S. Ahmed, Enas I. A. Mohamed, Elham Amin, Abeer S. Moawad, Mohamed Sadek Abdel-Bakky, Suliman A. Almahmoud, Naglaa Afifi

**Affiliations:** 1 Department of Pharmacognosy, Faculty of Pharmacy, Beni-Suef University, Beni-Suef, 62514 Egypt; 2 Department of Medicinal Chemistry and Pharmacognosy, College of Pharmacy, Qassim University, Buraydah, 51452 Saudi Arabia; 3 Department of Pharmacology and Toxicology, College of Pharmacy, Qassim University, Buraydah, 51452 Saudi Arabia; 4 Department of Pharmacology and Toxicology, Faculty of Pharmacy, Al-Azhar University, Cairo, 11751 Egypt

**Keywords:** *A*. *leucoclada*, NMR, GC–MS, COX, Anti-inflammatory, Molecular docking simulation

## Abstract

**Background:**

The plant kingdom has long been considered a valuable source for therapeutic agents, however, some plant species still untapped and need to be phytochemically and biologically explored. Although several *Atriplex* species have been investigated in depth, *A. leucoclada,* a halophytic plant native to Saudi Arabian desert, remains to be explored for its phytochemical content and biological potentials. Herein, the current study investigated the metabolic content and the anti-inflammatory potential of *A*. *leucoclada.*

**Methods:**

Powdered aerial parts of the plant were defatted with n-hexane then the defatted powder was extracted with 80% methanol. n-Hexane extract (ATH) was analyzed using GC–MS, while the defatted extract (ATD) was subjected to different chromatographic methods to isolate the major phytoconstituents. The structures of the purified compounds were elucidated using different spectroscopic methods including advanced NMR techniques. Anti-inflammatory activity of both extracts against COX-1 and COX-2 enzymes were examined in vitro. Molecular docking of the identified compounds into the active sites of COX-1 and COX-2 enzymes was conducted using pdb entries 6Y3C and 5IKV, respectively.

**Results:**

Phytochemical investigation of ATD extract led to purification and identification of nine compounds. Interestingly, all the compounds, except for 20-hydroxy ecdysone (1), are reported for the first time from *A. leucoclada*, also luteolin (6) and pallidol (8) are isolated for the first time from genus *Atriplex*. Inhibitory activity of ATD and ATH extracts against COX-1 and COX-2 enzymes revealed concentration dependent activity of both fractions with IC_50_ 41.22, 14.40 μg/ml for ATD and 16.74 and 5.96 μg/ml for ATH against COX-1 and COX-2, respectively. Both extracts displayed selectivity indices of 2.86 and 2.80, respectively as compared to 2.56 for Ibuprofen indicating a promising selectivity towards COX-2. Molecular docking study supported in vitro testing results, where purified metabolites showed binding affinity scores ranged from -9 to -6.4 and -8.5 to -6.6 kcal/mol for COX-1 and 2, respectively, in addition the binding energies of GC–MS detected compounds ranged from -8.9 to -5.5 and -8.3 to -5.1 kcal/mol for COX-1 and 2, respectively as compared to Ibuprofen (-6.9 and -7.5 kcal/mol, respectively), indicating high binding affinities of most of the compounds. Analysis of the binding orientations revealed variable binding patterns depending on the nature of the compounds. Our study suggested *A*. *leucoclada* as a generous source for anti-inflammatory agents.

**Supplementary Information:**

The online version contains supplementary material available at 10.1186/s12906-023-04281-5.

## Introduction

Inflammation is a self-defense mechanism that is triggered by pathogens, tissue injury, trauma or dysregulation of the normal physiological processes. Inflammatory response rises from the production of prostaglandins that is synthesized from the unsaturated arachidonic acid via cyclooxygenase (COX) enzymes [1, 2]. Cyclooxygenase enzymes are responsible for the formation of important biological mediators called prostanoids, including prostaglandins, prostacyclin and thromboxane. There are two known isoenzymes: COX-1 and COX-2. COX-1 is constitutively expressed in many tissues and is the predominant form in gastric mucosa and in kidney. COX-2 is not normally expressed in most cells, but elevated levels are found at sites of inflammation. Pharmacological inhibition of COX can provide relief signs of inflammation and pain [3].

Anti-inflammatory drugs include steroids and non-steroid drugs. Steroid drugs have some serious side effects such as osteoporosis and fractures, immunosuppression, myopathy, cardiovascular disease, glaucoma and cataracts, diabetes and hyperglycemia, psychiatric disturbances, gastrointestinal and dermatologic adverse effects. These adverse effects limit their utility and make them less popular to be used in inflammatory diseases compared with nonsteroidal drugs [4]. However, nonsteroidal drugs may have some side effects too such as bronchospasm, renal failure, thrombosis, and gastrointestinal bleeding [5]. To overcome these problems, herbal medicines and phytochemicals have been submitted to studies to identify and develop natural products that can be used as anti-inflammatory agents [6–8] or as a combinatorial therapy with these synthetic drugs [9].

The *Atriplex* genus (Amaranthaceae) constitutes herbaceous halophytes that include about 260 species distributed throughout the world, especially in the arid and semi-arid regions of Europe, Asia, Africa, Australia, and North America [10, 11]. Recent studies have shown that some species have high nutritional value and protein content and can be used as cereal grains as *A. hortensis* seeds [12]. Phytochemical investigations of some *Atriplex* species revealed various chemical constituents belonging to different chemical classes as: phenolics [13, 14], triterpenes, sterols [15], phytoecdysteroids [16, 17] and triterpene saponins [13, 15, 18, 19]. From biological point of view some species have been reported to have anti-inflammatory [20], antioxidants, anticholinestrase [13], antidiabetic [21], antimicrobial [22], hepatoprotective [23], immunomodulatory [14], analgesic, antipyretic, and, cytotoxic activities [24, 25].

*Atriplex leucoclada* Boiss. (English name: cut-leaf saltbush, orach, Arabic name: Ragal, رغل), is a low perennial shrub commonly growing in Saudi Arabian desert. This species has an agricultural importance in arid regions. It can adapt high salt habitats via different strategies [13, 26]. Reviewing the relevant literature little research was found discussing the metabolic content and/or the biological activity of *A. leucoclada*, where, one previous study reported the isolation of five triterpenoidal saponins and highlighted their molluscidal potential [27]. Accordingly, this study was designed to add more research about the phytochemical constituents and anti-inflammatory activity of *A. leucoclada.*

## Materials and methods

### General experimental procedures

NMR spectra were obtained on Bruker Avance III 400 MHz with BBFO Smart Probe and Bruker 400 MHz AEON Nitrogen-Free Magnet (Bruker AG, Switzerland) operating at 400 MHz for proton and 100 MHz for carbon. Data were analyzed using Topspin 3.1 Software (Bruker AG, Fallanden, Switzerland). 1D and 2D-NMR spectra (^1^H, ^13^C, HSQC and HMBC*)* were obtained using standard Bruker pulse programs. All deuterated solvents (CDCl_3_, CD_3_OD and pyridine-*d*_5_) for NMR measurement were obtained from (Cambridge Isotopes, USA). Column chromatography was performed using silica gel 60 (Fluka, St. Louis, MO, USA, particle size 0.063–0.2 mm, 70–230 mesh), polyamide-6 (50–160 μm), and Sephadex LH-20 (Sigma-Aldrich, Germany). Solvents used in chromatographic isolation of secondary metabolites were of analytical grade*; n*-hexane, dichloromethane (CH_2_Cl_2_), ethyl acetate (EtOAc), methanol (MeOH), and *n-*butanol (*n-*BuOH). Pre-coated silica gel 60 TLC plates used for the analysis of fractions and isolated compounds were purchased from Merck (Darmstadt, Germany). Visualization of the TLC plates was achieved with portable UV lamp (254 and 365 nm), AlCl_3_ and *p*-anisaldehyde′s spray reagent [28]

### Plant material

Aerial parts of *A. leucoclada* were collected in October 2020 from the Qassim area, Kingdom of Saudi Arabia. The plant identity was verified by Ibrahim Aldakhil, area botanical expert, Qassim, KSA. Voucher sample number QPP-103 was deposited at the College of Pharmacy, Qassim University, KSA.

### Preparation of extract

The dried aerial parts of *A. leucoclada* (700 g) were pulverized by a grinder and defatted with *n*-hexane (4 × 750 mL, at room temperature) to provide 1.7 g ATH extract. Afterwards, air-dried defatted powdered aerial parts were extracted with 80% methanol (4 × 1000 mL, at room temperature) to yield 45 g crude ATD extract.

### Chromatographic isolation of phytochemicals

The defatted fraction (30 g) was fractionated on polyamide-6 using H_2_O-MeOH gradient to obtain two main sub-fractions after TLC monitoring; (A-I and A-II); A-I (eluted by 10–30% MeOH in H_2_O) was purified on Sephadex LH-20 column using MeOH to obtain compound 1 (5.0 mg). A-II (eluted with 70–100% MeOH in H_2_O) was chromatographed on a silica gel column using gradient elution with CH_2_Cl_2_-MeOH as eluent in 5% increments to obtain five sub-fractions; A-IIa (100 mg, eluted with 5% MeOH in CH_2_Cl_2_), A-IIb (40 mg, eluted with 15% MeOH in CH_2_Cl_2_), A-IIc (70 mg, eluted with 15% MeOH in CH_2_Cl_2_), A-IId (20 mg, eluted with 20% MeOH in CH_2_Cl_2_), A-IIe (25 mg, eluted with 25–30% MeOH in CH_2_Cl_2_). A-IIa was purified on Sephadex LH-20 using MeOH to afford two sub-fractions; the first one was purified on Sephadex LH-20 column using MeOH as eluent to obtain compound 2 (6.0 mg), the other was chromatographed on a silica gel column using mixtures of n-hexane–EtOAc as eluent in 5% increments to obtain mixture of compound 3 &4 (10.0 mg) and compound 5 (15.0 mg), respectively. A-IIb, A-IId and A-IIe were separately filtered through Sephadex LH-20 column using MeOH as eluent to obtain compounds 6 (8.0 mg), 8 (20.0 mg) & 9 (7.0 mg); respectively. A-IIc was recrystallized to obtain compound **7** (25 mg).

### Gas chromatography–mass spectrometry analysis

GC–MS system: thermo scientific trace 1310 gas chromatograph attached with ISQ LT single quadrupole mass spectrometer. Column used for separation was db5-ms, 30m; 0.25 mm id (J&W scientific) with temperature program; 40°c (3 min)—280°c (5 min) at 5°c/min. -290°c (1 min) at 7.5°c/min. Ionization mode: EI. Ionization voltage: 70eV. Detector temperature: 300°c. Injector temperature was adjusted at 200°c. Helium was used as a carrier gas at 1 ml/min flow rate. identification of components was based on Willey and NIST mass spectral data base [29].

### In vitro determination of COX-1 and COX-2 enzymatic activity

COX-1 and COX-2 inhibition assays are based on the detection of the florescence produced by prostaglandin G2 (i.e., the intermediate product produced by the COX-1 and 2 enzymes). The assay was performed by using COX-1 inhibitor screening kit (#K548-100, BioVision Inc.) and COX-2 inhibitor screening kit (#K547-100, BioVision Inc.) to measure in vitro COX-1 and COX-2 enzymatic activities respectively. Ibuprofen was used as a positive control. Samples and control were used at different concentrations: 0.01–100 ug/ml. According to the manufacturer’s instructions [2, 30], 10 μL of samples or Ibuprofen was added to each well, and 80 μL of reaction master mix was prepared (76 μL of COX buffer assay, 1 μL COX probe, 2 μL diluted COX cofactor, 1 μL COX-1 or COX-2) and added to each well, and the fluorescence was measured kinetically at (Ex/Em = 535/587 nm) at 25°C for 5–10 min. The experiments were performed in triplicate. The relative percentage of inhibition of COX-1 and COX-2 was calculated according to the following Equation:

$$\%\;\mathrm R\mathrm e\mathrm l\mathrm a\mathrm t\mathrm i\mathrm v\mathrm e\;\mathrm i\mathrm n\mathrm h\mathrm i\mathrm b\mathrm i\mathrm t\mathrm i\mathrm o\mathrm n=\left[\left(\mathrm{Absorbance}\;\mathrm{of}\;\mathrm{EC}-\mathrm{Absorbance}\;\mathrm{of}\;\mathrm S\right)/\mathrm{Absorbance}\;\mathrm{of}\;\mathrm{EC}\right]\times100$$

### Statistical analysis

All measurements were performed in triplicate and results were expressed as mean ± standard deviation (SD).

### In silico studies

Isolated metabolites from defatted methanol (ATD) extract as well as compounds detected during GC–MS analysis of *n*-hexane (ATH) extract were docked into the active sites of COX-1 and COX-2 enzymes using pdb entries; 6Y3C [31] and 5IKV [32], respectively that were retrieved from Protein Data Bank (https://www4.rcsb.org/). Structures of all compound were downloaded from PubChem [33] [July, 2023] and their energies were minimized using Chem Bio 3D (Chem Bio Office Ultra 12.0 suite). Docking studies were performed using Autodock Vina in Pyrx [34]. XYZ coordinates were set as; 6Y3C: -30.39, -44.13, 7.77; 5IKV: 166.48, 183.18, 186.96 BIOVIA Discovery Studio visualizer v21.1.0.20298 (Dassault systems Biovia Corp., San Diego, CA, USA) and Pymol software [35] were used to visualize and analyze the docked ligand poses.

## Results

### Structural determination of isolated compounds

**Fig. 1:**
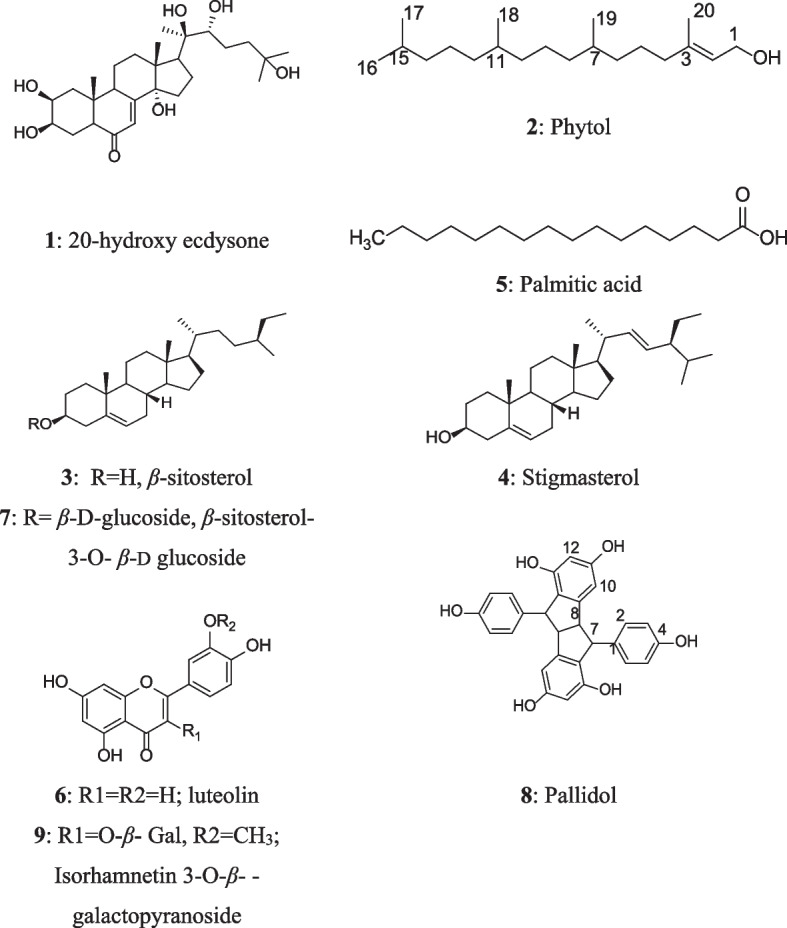
Chemical structures of the isolated compounds from the defatted methanolic extract (ATD) of *A. leucoclada*

Chromatographic fractionation of defatted fraction of aerial parts (ATD) led to the isolation and characterization of nine known compounds. The structures of the isolated compounds were identified upon spectral data analysis (Spectroscopic data of compounds were shown in supplementary materials) and confirmed by comparison with those published in the literature (Fig. 1) as: 20-hdroxy ecdysone (1) [36], phytol (2) [37], *β*-sitosterol (3) [38], stigmasterol (4) [38], palmitic acid (5) [39], luteolin (6) [38, 40], *β*-sitosterol-3-*O*-*β*-d-glucopyranoside (7) [40, 41], pallidol (8) [42, 43] and isorhamnetin 3-*O*-*β*-galactopyranoside (9) [44, 45].

### Gas Chromatography–Mass Spectrometry (GC–MS) analysis

**Fig. 2:**
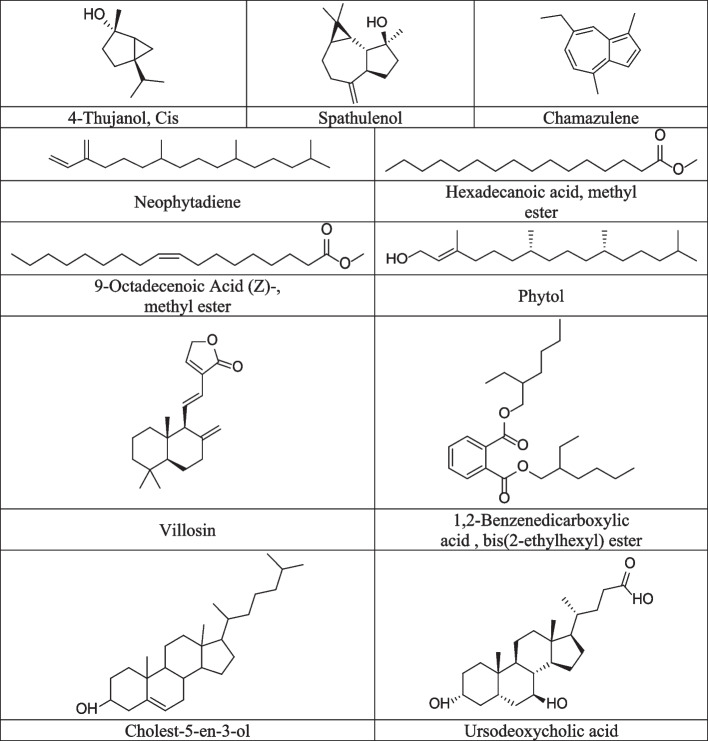
The most characteristic compounds identified in *n*-hexane (ATH) extract of *A. leucoclada* using GC–MS analysis

The chemical composition of the ATH extract of *A. leucoclada* was investigated using gas chromatography-mass spectrometry (GC–MS) analysis. A total of 27 metabolites were identified (Table 1); accounting for 85.97% of the total compounds. The identified compounds (Fig. 2) belong to three main classes including terpenoids, fatty acids and their derivatives and steroids (31.27, 21.18, and 20.25%; respectively). Other identified compounds included straight-chain hydrocarbons and derivatives (5.59%), 1,2-benzene dicarboxylic acid, bis(2-ethylhexyl) ester, 1,4-benzenediol, 2-(1,1-dımethylethyl)-5-(2-propenyl)-, and chamazulene (5.43, 1.11, and 1.14%). Phytol and cholest-5-en-3-ol (Fig. 2) were the main components (21.24, 12.50%; respectively).

**Table 1:** Chemical profile of *n*-hexane extract (ATH) of *A. leucoclada* using GC–MS analysis

NO	Compound	Chemical class	M.F	M.Wt	R.T (m)	Area (%)
**1. **	4-Thujanol, Cis-(± .)	Bicyclic monoterpene alcohol	C_10_H_18_O	154	9.08	0.96
**2. **	1,4-Benzenediol, 2-(1,1-dimethylethyl)-5-(2-propenyl)-	Hydroquinone	C_13_H_18_O_2_	206	17.31	1.11
**3. **	Spathulenol	Sesquiterpene	C_15_H_24_O	220	18.82	0.77
**4. **	Neophytadiene	Diterpene	C_20_H_38_	278	24.50	3.48
**5. **	2-Pentadecanone, 6,10,14-trimethyl-	Ketone	C_18_H_36_O	268	24.60	3.51
**6. **	Chamazulene	Azulene derivative	C_14_H_16_	184	25.00	1.14
**7. **	13-Heptadecyn-1-ol	Long-chain fatty alcohol	C_17_H_32_O	252	25.37	1.17
**8. **	Hexadecanoic acid, methyl ester	Fatty acid ester	C_17_H_34_O_2_	270	26.30	6.45
**9. **	9-Octadecenoic acid (*Z*)	Fatty acid	C_18_H_34_O_2_	282	27.63	0.65
**10. **	7,10-Octadecadienoic acid, methyl ester	Fatty acid ester	C_19_H_34_O_2_	294	29.43	2.24
**11. **	9-Octadecenoic Acid (*Z*)-, methyl ester	Fatty acid ester	C_19_H_36_O_2_	296	29.57	4.01
**12. **	Phytol	Acyclic diterpene alcohol	C_20_H_40_O	296	29.77	21.24
**13. **	Heptadecanoic acid, 16-methyl-, methyl ester	Fatty acid ester	C_19_H_38_O_2_	298	30.09	1.86
**14. **	[1,1′-Bicyclopropyl]-2-octanoic acid, 2′-hexyl-, methyl ester	Fatty acid ester	C_21_H_38_O_2_	322	30.64	0.84
**15. **	2-Hydroxy-3-[(9e)-9-octadecenoyloxy]propyl (9e)-9-octadecenoate	Fatty acid ester	C_39_H_72_O_5_	620	33.84	1.09
**16. **	Villosin	Diterpene	C_20_H_28_O_2_	300	35.21	1.13
**17. **	1-Heptatriacotanol	Alcohol	C_37_H_76_O	536	36.24	0.91
**18. **	9,19-Cyclolanostan-3-ol, 24,24-epoxymethano-, acetate	Steroid	C_33_H_54_O_3_	498	36.35	0.81
**19. **	Ethyl iso-allocholate	Steroid	C_26_H_44_O_5_	436	36.63	1.15
**20. **	1,2-Benzenedicarboxylic acid, bis(2-ethylhexyl) ester	Benzenedicarboxylic acid	C_24_H_38_O_4_	390	36.75	5.43
**21. **	9-Octadecenoic acid, 1,2,3-propanetriyl ester, (*E*, *E*, *E*)-	Fatty acid ester	C_57_H_104_O_6_	884	39.39	1.12
**22. **	Glycidyl oleate	Fatty acid ester	C_21_H_38_O_3_	338	39.55	0.94
**23. **	Trilinolein	Triacylglycerol	C_57_H_98_O_6_	878	40.82	1.98
**24. **	Rhodopin	Carotenoid (tetraterpenoid)	C_40_H_58_O	554	41.02	3.69
**25. **	*β*- Sitosterol	Steroid	C_29_H_50_O	414	43.12	2.07
**26. **	Cholest-5-en-3-ol	Steroid	C_27_H_46_O	414	43.79	12.50
**27. **	Ursodeoxycholic acid	Steroid	C_24_H_40_O_4_	392	45.34	3.72
**Terpenoids**						31,27
**Steroids**						20.25
**Fatty acids and fatty acids derivatives**						21.18
**Straight-chain hydrocarbons and derivatives**						5.59
**Others**						7.68
**Total identified compounds %**						85.97

### In vitro determination of COX-1 and COX-2 inhibitory activity

**Fig. 3:**
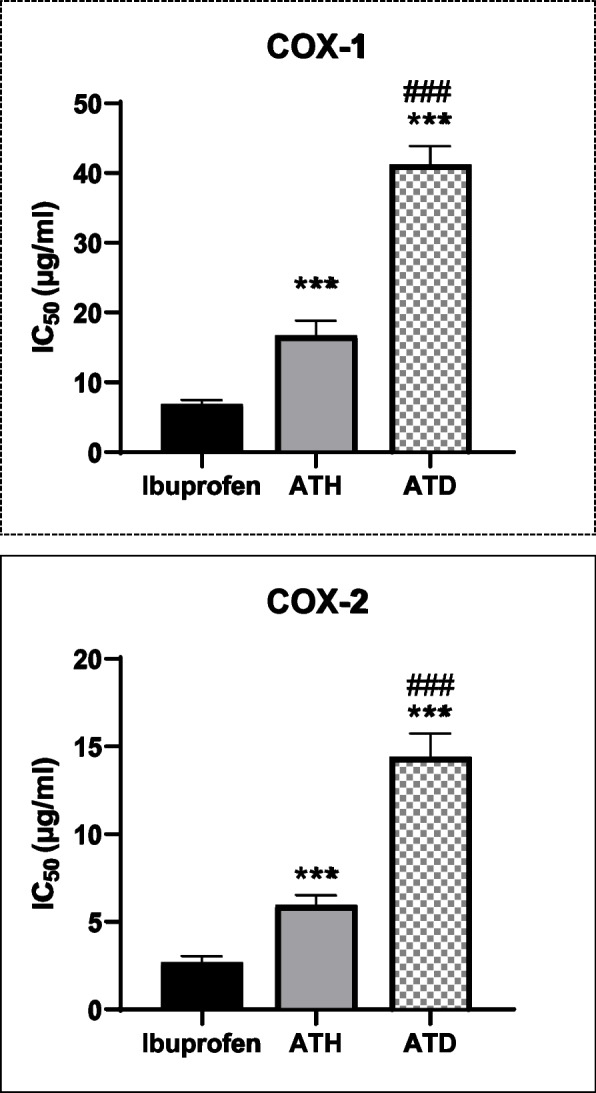
The in vitro inhibitory effect of *n*-hexane (ATH) and defatted methanol (ATD) extracts of *A. leucoclada* against COX-1 (**A**) and COX-2 (**B**) enzymes using Ibuprofen as a positive control. Data in the figures expressed mean ± SEM (n = 3). ^***^P < 0.001 consider statistically significant compared to ibuprofen. ^###^*P* < 0.001 consider statistically significant compared to ATH group using one way ANOVA followed by Tukey’s post hoc test

In the present study, inhibitory effects of the defatted methanolic extract (ATD) and *n*-hexane (ATH) extract against COX-1 and COX-2 enzymes were examined in vitro. The results (Fig. 3A and B) showed that the tested extracts displayed inhibition of COX-1 and COX-2 enzymes in a concentration dependent manner being more selective towards COX-2 enzyme. Where IC_50_ of ATD were 41.22, 14.4 μg/mL and of ATH were 16.74 and 5.96 μg/mL, against COX-1 and COX-2, respectively while ibuprofen IC_50_ was 6.88 and 2.68, respectively (Table 2).

**Table 2:** IC_50_ (μg/ml) values against COX-1 and COX-2 for *n*-hexane and defatted methanol extracts from *A. Leucoclada* and the COX-1/COX-2 selectivityy index

	**COX-1**	**COX-2**	**SI**
Ibuprofen	6.88 ± 0.27	2.68 ± 0.1	2.56
ATH	16.74 ± 0.94^***^	5.96 ± 0.22^***^	2.81
ATD	41.22 ± 1.18^***, ###^	14.4 ± 0.54^***, ###^	2.86

Defatted methanol extract (ATD); *n*-hexane extract (ATH); Cyclooxygenase (COX); The half of the inhibitory concentration (IC_50_). Selectivity index (SI) = IC_50_ COX-1/IC_50_ COX-2. Data in the Table expressed mean ± SEM (n = 3). Where ^***^P < 0.001 consider statistically significant compared to ibuprofen and ^###^*P* < 0.001 consider statistically significant compared to ATH group using one-way ANOVA followed by Tukey’s post hoc test

### In silico studies

Isolated compounds from ATD extract along with compounds identified by GC–MS profiling of ATH were subjected to molecular docking with COX-1 and COX-2 proteins. The majority of the compounds manifested high binding affinities and good binding interactions. The docked purified metabolites from ATD showed scores for COX-1 and COX-2 ranged from -9 to -6.4 and -8.5 to -6.6 kcal/mol, respectively, while docking scores of metabolites that identified by GC–MS in ATH ranged from -8.9 to -5.5 and -8.3 to -5.1 kcal/mol, respectively (Table 3), compared with binding scores of the drug reference; Ibuprofen for COX-1 and 2 (-6.9 and -7.5 kcal/mol, respectively). The flavone; luteolin exhibited the highest binding affinity to COX-2 (-8.5 kcal/mol) followed by the steroids; *β*-sitosterol-3-O-*β*-D-glucoside (-8.4 kcal/mol), stigmasterol (-8.4 kcal/mol), 9,19-Cyclolanostan-3-ol, 24,24-epoxymethano-, acetate (-8.3 kcal/mol), and 20-hydroxy ecdysone (-8.1 kcal/mol). Among terpenoids, the diterpene villosin and the tetraterpene rhodopsin revealed utmost binding affinities (-7.7 and -7.6 kcal/mol, respectively), while 2-Hydroxy-3-[(9e)-9-octadecenoyloxy]propyl (9e)-9-octadecenoate unveiled the best score (-7.1 kcal/mol) among other fatty acid esters.

**Table 3:** Docking scores (kcal/mol) of isolated compounds from defatted methanolic (ATD) extract and compounds detected by GC–MS in *n*-hexane (ATH) extract of *A. leucoclada* against cyclooxygenase enzymes

No	**Compound**	**COX-1**	**COX-2**
Compounds isolated from ATD extract of *A. leucoclada*			
1	20-hydroxy ecdysone	-8.0	-8.1
4	Stigmasterol	-9.0	-8.4
5	Palmitic acid	-6.4	-6.6
6	Luteolin	-8.2	-8.5
7	*β*-sitosterol-3-O-* β*-D glucoside	-8.1	-8.4
8	Pallidol	-8.5	-8.1
9	Isorhamnetin3-O-*β*-D-galactopyranoside	-7.7	-7.8
Compounds detected in ATH extract of *A. leucoclada*			
1	4-Thujanol, Cis	-5.8	-5.4
2	1,4-Benzenediol, 2-(1,1-dimethylethyl)-5-(2-propenyl)-	-6.4	-6.3
3	Spathulenol	-5.9	-6.5
4	Neophytadiene	-6.5	-5.5
5	2-Pentadecanone, 6,10,14-trimethyl-	-6.2	-7.0
6	Chamazulene	-7.7	-7.6
7	13-Heptadecyn-1-ol	-6.4	-6.5
8	Hexadecanoic acid, methyl ester	-6.1	-6.1
9	9-Octadecenoic acid (*Z*)	-5.5	-6.7
10	7,10-Octadecadienoic acid, methyl ester	-7.3	-6.0
11	9-Octadecenoic Acid (*Z*)-, methyl ester	-7.1	-6.1
12	Phytol	-6.2	-5.6
13	Heptadecanoic acid, 16-methyl-, methyl ester	-6.5	-6.4
14	[1,1′-Bicyclopropyl]-2-octanoic acid, 2′-hexyl-, methyl ester	-6.4	-5.6
15	2-Hydroxy-3-[(9e)-9-octadecenoyloxy]propyl (9e)-9-octadecenoate	-6.5	-7.1
16	Villosin	-7.3	-7.7
17	1-Heptatriacotanol	-6.5	-5.2
18	9,19-Cyclolanostan-3-ol, 24,24-epoxymethano-, acetate	-8.4	-8.3
19	Ethyl iso-allocholate	-7.4	-7.4
20	1,2-Benzenedicarboxylic acid, bis(2-ethylhexyl) ester	-5.7	-6.3
21	9-Octadecenoic acid, 1,2,3-propanetriyl ester, (*E*, *E*, *E*)-	-6.1	-6.7
22	Glycidyl oleate	-7.3	-5.1
23	Trilinolein	-6.6	-6.7
24	Rhodopin	-8.0	-7.6
25	*β*-sitosterol	-8.9	-7.5
26	Cholest-5-en-3-ol	-7.6	-7.4
27	Ursodeoxycholic acid	-7.4	-7.4

## Discussion

Reviewing the relevant literature several studies were found reporting diverse chemical structures of the metabolic contents of some *Atriplex* species. While, in regards of *A. leucoclada,* previous investigations were not sufficient to describe the chemical profile of the plant. To achieve this purpose, the chemical composition of both defatted methanol (ATD) and hexane (ATH) extracts were investigated. The current findings showed that all purified compounds, except for compound (1), were isolated for the first time from *A. leucoclada* [46]. Furthermore, compounds (6) and (8) were isolated for the first time from this genus. Compound (1) was previously obtained from other *Atriplex* species as *A. inflata* and *A. nummularia* [16, 17], compounds (3) and (4) were previously reported in *A. stocksii* [15], compound (**7**) in *A. canescens* [47] and compound (9) in *A. inflate* [46], while compound (2) and (5) were identified by GC–MS only in the methanol extract of *A. halimus* [48, 49].

Interestingly, this study is the first to report GC–MS investigation of *A. leucoclada*. The current GC–MS analysis results enabled the tentative qualitative identification of numerous phytochemicals in ATH. The compounds identified have been interpreted as given in Table 1. Oxygenated and non-oxygenated hydrocarbons, alcohols, phenolics, steroidal and terpenoidal compounds were identified. Among the isolated compounds: phytol (21.24%) and cholest-5-en-3-ol (12.50%) are the major detected compounds. These results were similar to those reported for GC–MS analysis for *A. lindleyi* Moq [50].

Based upon the above recorded results, the anti-inflammatory activity of *A. leucoclada* may be attributed to its content of palmitic (5) and *β*-sitosterol (3) that were previously reported to reduce expression of COX- 1 and COX-2 [51–53]. Moreover, 20-hdroxy ecdysone (1) [54], stigmasterol (4) [55], luteolin (6) [56], *β*-sitosterol-3-O-*β*-D-glucopyranoside (**7**) [57], and isorhamnetin 3-O-*β*-galactopyranoside (9) [58] were also reported to suppress COX-2 expression. Also, computational study on phytol (2) indicated its efficient interaction with COX-1 and 2 enzymes [59]. Concerning pallidol (8), a resveratrol dimer, it was reported to have weak activity against COX enzymes [60, 61]

In regard of GC–MS results, the high anti-inflammatory activity of the ATH fraction may be attributed to the synergistic effect of certain compounds e.g. 2-hexadecen-1-ol [62] and hexadecanoic acid, methyl ester [63]. Also, ursodeoxycholic acid was reported to show COX-2 inhibition [64]. From another point of view, neophytadiene significantly inhibited NO production and inflammatory cytokines TNF-α, IL-6 and IL-10 both in vitro and in vivo [65, 66], azulene derivative reverses osteoarthritic inflammation through regulation of matrix metalloproteinases and NF-kβ pathway in in vitro and in vivo models [67] and villosin exerted inhibitory effects against NO production with IC_50_ = 15.5 μM [68].

Most of the compounds identified in the ATD or ATH extracts were previously reported to be more selective for COX-2. This is compatible with the current results that indicated high selectivity towards COX-2 enzyme. As selective COX-2, anti-inflammatory agents have minimum GIT side effects, and so they are more appreciated as per safety concern. Accordingly, the current findings acknowledge the metabolic content of *A. leucoclada* as a rich mixture of chemical entities that have promising potential of anti-inflammatory activity with limited side effects.

**Fig. 4:**
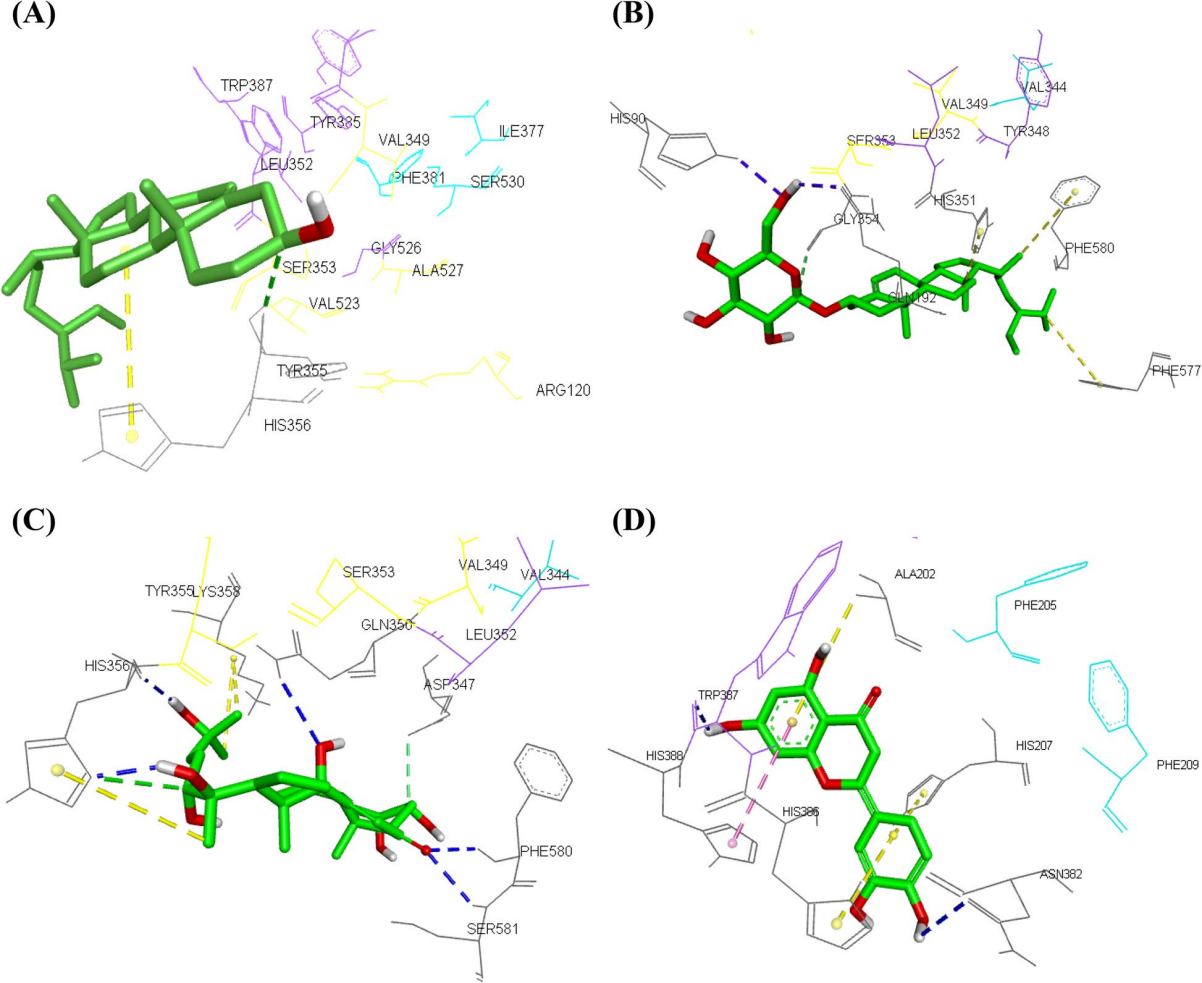
3D docking view of *β*-sitosterol (**A**), *β*-sitosterol-3-O-*β*-D-glucopyranoside (**B**), 20-hydroxy-ocdysone (**C**), and luteolin (**D**) in the active site of COX-2. Labeled residues that constitute the proximal (yellow), central (magenta), and distal (cyan) binding pockets and other interacting residues in the active site (gray) are depicted in line models. Ligands are represented by green stick model. Hydrogen bond (blue), π-π stacking (pink), alkyl (yellow), and carbon-hydrogen bond (green) interactions are depicted in dashed lines

It is already stated that, computational studies played an effective role in drug development as it can provides a fast, cheap and easy method for expectation of the possible promising bioactive drugs. Herein, we examined the binding affinity of isolated (from ATD extract) as well as GC–MS identified compounds (in ATH extract) in this plant with key anti-inflammatory targets. Analysis of the binding with the two proteins COX-1 and COX-2 revealed variable binding patterns depending on the nature of the compounds. Steroids that constitute 44.44% of isolated compounds from ATD extract and 20.25% of detected compounds in ATH extract exhibited docking score with COX-2 ranged from -8.4 to -7.4 kcal/mol. During this study, we explored some data that can be gleaned from analysis of steroids conformation and interactions at the cyclooxygenase active site. Previous studies that examined the binding of the substrate “arachidonic acid” into COX active site suggested that the active site could be viewed as including three parts; proximal, central, and distal binding pockets and that the distal and proximal binding pockets are important for stabilization of the substrate, while the central part that contains the catalytic Tyr385 is the place where substrate is transformed into PGG_2 _[69]. Analysis of our docking results unveiled that steroids occupied the proximal binding pocket and may extends towards the central binding pocket. It was noted that presence of more hydrophilic groups at C-3 may contribute to the increased binding affinity as exemplified in *β*-sitosterol3-O-*β*-D-glucopyranoside (-8.4 kcal/mol) with glucosylation at C-3 where it exhibited flat inverted conformation compared to that of *β*-sitosterol, furthermore, one of the sugar hydroxyl groups formed two hydrogen bonds with His90 and Gln192 in the side pocket, a pocket that was generated in COX-2 due to Ile523 mutation in COX-1 to the smaller Val523 in COX-2 [69] (Fig. 4B), while *β*-sitosterol (-7.4 kcal/mol) exhibited only covalent bonds with Tyr355 at the constriction located near the entrance of the active site and with His90, Ser353, and His356 (Fig. 4A). Similarly, presence of carbonyl group at C-6 and hydroxyl groups at C-14, C-20, C-22, and C-25 as in the case of 20-hydroxyecdysone (-8.4 kcal/mol) may play a role in improving its binding affinity via formation of two hydrogen bonds between His356 and OH groups at C-20 and C-25, respectively and oxygen of the carbonyl group at C-6 exhibited two hydrogen bonds with Phe 580, and Ser581, in addition to hydrogen bonding between hydroxyl group at C-14 and Gln350 (Fig. 4C). Additionally, the current findings highlighted the flavonoid luteolin that was one of the lead metabolites exhibiting the highest binding affinity to COX-2 (-8.5 kcal/mol); analysis of the binding orientation of luteolin revealed that it formed one hydrogen bond with Trp387 in the catalytic region of the active site and another hydrogen bond with Asn382 in addition to π-π stacking with His388 and three π-alkyl interactions with Ala202, His207, and His386 (Fig. 4D).

## Conclusion

The current study presented a detailed chromatographic exploration of *A*. *leucoclada* with successful isolation of nine compounds, eight of which were isolated for the first time from this species, moreover, GC–MS analysis identified the non-polar components of the hexane extract of the plant. These findings will add to chemical profile of this species, as well as to *Atriplex* genus. Additionally, COX-1 and COX-2 inhibitory activity testing noted the *n*-hexane and defatted methanol extracts for selective inhibitory activity against COX-2 enzyme. The molecular docking studies revealed high binding affinities and good binding interactions of most of the compounds with binding scores ranged from -8.5 to -6.6 kcal/mol for isolated compounds from ATD and -8.3 to -5.1 kcal/mol for compounds identified by GC–MS in ATH. Accordingly, *A*. *leucoclada* is suggested as a valuable source of safe anti-inflammatory agents. Future studies are recommended for evaluating the anti-inflammatory potential of the highlighted metabolites whether, alone or in combination with commercially used anti-inflammatory drugs.

### Supplementary Information

**Additional file 1: Figure S1.** 2D Docking poses of *β*-sitosterol (A), *β*-sitosterol-3-O-*β*-D-glucopyranoside (B), 20-hydroxy-ocdysone (C), and luteolin (D) in the active site of COX-2.

## Data Availability

All obtained data have been included in the manuscript.
