# Supplementary material for: Phytochemical investigation and anti-inflammatory potential of Atriplex leucoclada Boiss
Source: BMC Complement Med Ther. 2023 Dec 16;23:464. doi: 10.1186/s12906-023-04281-5 (PMC10725009; doi:10.1186/s12906-023-04281-5)
Supplement: Supplementary file 1 — Additional file 1: Figure S1. 2D Docking poses of β-sitosterol (A), β-sitosterol-3-O-β-D-glucopyranoside (B), 20-hydroxy-ocdysone (C), and luteolin (D) in the active site of COX-2. [file 12906_2023_4281_MOESM1_ESM.docx]

Phytochemical investigation and anti-inflammatory potential of *Atriplex leucoclada* Boiss

Hayam S. AHMED^1*^†, Enas I. A. Mohamed^1^†, Elham AMIN^2^, Abeer S. MOAWAD^1^, Mohamed ABDELBAKKY^3,4^, Suliman A. ALMAHMOUD^2^, Naglaa AFIFI^1^ ^1^Department of Pharmacognosy, Faculty of Pharmacy, Beni-Suef University, Beni-Suef 62514, Egypt

^2^Department of Medicinal Chemistry and Pharmacognosy, College of Pharmacy, Qassim University, Buraydah 51452, Saudi Arabia

^3^Department of Pharmacology and Toxicology, College of Pharmacy, Qassim University, Qassim 51452, Saudi Arabia

^4^Department of Pharmacology and Toxicology, Faculty of Pharmacy, Al-Azhar University, Cairo 11751, Egypt

* Corresponding author: Hayam011169@pharm.bsu.edu.eg

† Authors with equal contributions

Contents

[Spectral Identification of isolated compounds: 2](#_Toc140837069)

[Figure S1: 2D Docking poses of *β*-sitosterol (A), *β*-sitosterol-3-O-*β*-D-glucopyranoside (B), 20-hydroxy-ocdysone (C), and luteolin (D) in the active site of COX-2. 5](#_Toc140837070)

[References: 6](#_Toc140837071)

# Spectral Identification of isolated compounds:

**20-hdroxy ecdysone (1):** ^1^H-NMR (400 MHz, MeOD-*d*_4_); δ_H_ 1.4, 1.8 (4H, d, *J*= 3.2, 12.0 Hz, H-1, H-24), 3.86 (1H, d, *J*=10.4 Hz, C-2), 3.97 (1H, br s, H-3), 1.65, 1.74 (2H, H-4), 2.37 (2H, dd, *J*= 16, 5.6, Hz, H-5, H-17), 5.81 (1H, d, *J*= 2, Hz, H-7), 3.14 (1H, t , *J* = 9.2, Hz, H-9), 1.74, 1.97 (4H, H-11, H-16), 1.89, 2.10 (2H, H-12), 1.62, 1.97 (2H, H-15), 3.35 (1H, s, H-22), 1.3, 1.67 (2H, d, H-23).(5×3H); 0.89 (H-18), 0.97 (H-19), 1.19 (H-21), 1.20 (H-26) and 1.20 (H-27). DEPT-Q NMR (101 MHz, MeOD-*d*_4_); δ_C_ 37.4 (C-1), 68.6 (C-2), 68.5 (C-3), 32.8 (C-4), 51.7 (C-5), 206.4 (C-6), , 122.1 (C-7), 168.0 (C-8), 35.1 (C-9), 39.2 (C-10), 21.5 (C-11), 32.5 (C-12), overlapped with solvent(C-13), 85.2 (C-14), 31.7 (C-15), 21.5 (C-16), 50.5 (C-17), 18.04 (C-18), 24.4 (C-19), 77.9 (C-20), 21.0 (C-21), 78.4 (C-22), 27.3 (C-23), 42.3 (C-24), 71.3 (C-25), 29.0 (C-26), 29.7 (C-27) [1].

**Phytol (2):** ^1^H-NMR (400 MHz, pyridine-*d*_5_); major signals δ_H_: 4.47 (2H, d, *J*=7.2 Hz, H-1), 5.76 (1H, td, *J*=6.8, 1.2 Hz), (5×3H); 0.94 (H-16), 0.95 (H-17), 0.90 (H-18), 0.89 (H-19) and 1.68 (H-20).DEPT-Q NMR (101 MHz, Pyridine-*d*_5_); δ_C_ 59.2 (C-1), 126.5 (C-2), 145.5 (C-3), 40.5 (C-4), 25.9 (C-5), 38.1 (C-6), 33.3 (C-7), 37.9 (C-8), 25.14 (C-9), 37.3 (C-10), 33.4 (C-11),38.02 (C-12), 25.5 (C-13), 39.9 (C-14), 28.5 (C-15), 23.2 (C-16), 23.1 (C-17), 20.3 (C-18), 20.2 (C-19), 16.5 (C-20) [2].

***β*-sitosterol (3):** ^1^H-NMR (400 MHz, Pyridine-*d*_5_); Major signals; δ_H_: 5.45 (br s, H-6), 3.86 (m, H-3), (6×3H); 1.08 ((H-19), 1.02 (H-21), 0.9 (H-26), 0.89 (H-27), 0.87 (H-29), and 0.72 (H-18). DEPT-Q NMR (101 MHz, pyridine-*d*_5_) ); δ_C_ 38.2 (C-1), 30.4 (C-2), 71.7 (C-3), 43.9 (C-4), 142.4 (C-5), 121.6 (C-6), 32.6 (C-7), 32.6 (C-8), 50.0 (C-9), 37.3 (C-10), 21.8 (C-11), 40.4 (C-12), 42.9 (C-13), 57.3 (C-14), 24.9 (C-15), 28.9 (C-16), 56.7 (C-17), 12.4 (C-18), 20.0 (C-19), 36.9 (C-20), 19.4 (C-21), 34.6 (C-22), 26.8 (C-23), 46.5 (C-24), 29.9 (C-25), 20.4 (C-26), 19.6 (C-27), 23.80 (C-28) and 12.6 (C-29) [3].

**Stigmasterol (4):** ^1^H-NMR (400 MHz, CDCl_3_): δH 5.45 (br s, H-6), 5.23 (m, H-22), 5.103 (m, H-23), 3.86 (m, H-3), (6×3H); 1.08 ((H-19), 1.02 (H-21), 0.9 (H-26), 0.89 (H-27), 0.87 (H-29), and 0.72 (H-18). DEPT-Q NMR (101 MHz, Pyridine-*d*_5_) ); δ_C_ 38.2 (C-1), 30.4 (C-2), 71.7 (C-3), 43.9 (C-4), 142.4 (C-5), 121.6 (C-6), 32.6 (C-7), 32.6 (C-8), 50.0 (C-9), 37.3 (C-10), 21.8 (C-11), 40.4 (C-12), 42.9 (C-13), 57.3 (C-14), 24.9 (C-15), 28.9 (C-16), 56.7 (C-17), 12.4 (C-18), 20.0 (C-19), 36.9 (C-20), 19.4 (C-21), 139.2 (C-22), 129.8 (C-23), 46.5 (C-24), 29.9 (C-25), 20.4 (C-26), 19.6 (C-27), 23.80 (C-28) and 12.6 (C-29) [3].

**Palmitic acid (5):** ^1^H NMR (400 MHz, CDCl_3_) δ 2.34 (2H, t, *J*= 7.6, H-2), 1.63 (2H, m, H-3), 0.88 (3H, t, *J*= 7.6, H-16) [4].

**Luteolin (6):** ^1^H NMR (400 MHz, CDCl3), *δ*_H_ (ppm): 6.53 (1H, s, H-3), 6.20 (1H, d, *J*=2.4 Hz, H-6), 6.43 (1H, d, *J*=2.4 Hz, H-8), 7.36 (1H, overlapped, H-2*'*), 6.89 (1H, d, *J*= 9.2 Hz, H-5′), 7.38 (1H, overlapped, H-6′) [3, 5].

***β*-sitosterol-3-*O*-*β*-D-glucopyranoside (7):** ^1^H-NMR (400 MHz, Pyridine-*d*_5_); major signals δ_H_: 5.36 (br s not t, *J*=2.4 Hz, H-6), 5.07 (d, *J*=7.2 Hz, anomeric proton), 4.60-3.99 (6H, sugar protons), 3.99 (m, H-3), (6×3H); 1.01 (H-19), 0.95 (H-21), 0.93 (H-26), 0.91 (H-27), 0.87 (H-18), and 0.68 (29). DEPT-Q NMR (101 MHz, Pyridine-*d*_5_); δ_C_: 37.8 (C-1), 30.6 (C-2), 78.5 (C-3), 39.7 (C-4), 141.3 (C-5), 122.3 (C-6), 32.5 (C-7), 32.4 (C-8), 50.7 (C-9), 37.2 (C-10), 21.7 (C-11), 40.3 (C-12), 42.8 (C-13), 57.2 (C-14), 24.8 (C-15), 28.9 (C-16), 56.6 (C-17), 12.3 (C-18), 19.6 (C-19), 36.7 (C-20), 19.4 (C-21), 34.6 (C-22), 26.8 (C-23), 46.4 (C-24), 29.9 (C-25), 20.3 (C-26), 19.8 (C-27), 23.7 (C-28), 12.5 (C-29), 102.9 (C-1′), 75.7 (C-2′), 80.0 (C-3′), 72.1 (C-4′), 78.9 (C-5ʹ), and 63.2 (C-6ʹ) [5, 6].

**Pallidol (8):** ^1^H-NMR (400 MHz, MeOD-*d*_4_) δ_H_: 6.94 (d, *J* = 8 Hz, 4H, H-2, H-6, H-2′, and H-6′), 6.68 (d*, J =* 8.4 Hz, 4H, H-3, H-5, H-3′ and H-5′), 6.55 (brs, 2H, H-10 and H-10′), 6.13 (brs, 2H, H-12 and H-12′), 4.49 (s, 2H, H-7 and H-7′), and 3.75 (s, 2H, H-8 and H-8′). DEPT-Q NMR (101 MHz, MeOD-*d*_4_) δ_C_: 159.2 (C-11 and C-11′), 156.2 (C-4 and C-4′), 155.4 (C-13 and C-13′), 150.7 (C-9 and C-9′), 138.4 (C-1 and C-1′), 129.1 (C-2, C-6, C-2′, and C-6′), 123.8 (C-14 and C-14′), 115.9 (C-3, C-5, C-3′, and C-5′), 103.3 (C-10 and C-10′), 102.5 (C-12 and C-12′), 60.9 (C-8 and C-8′), and 54.6 (C-7 and C-7′) [7, 8].

**Isorhamnetin 3-*O*-*β*-galactopyranoside (9):** ^1^H NMR (400 MHz, MeOD-*d*_4_), *δ*_H_: 6.19 (1H, d, *J*=2 Hz, H-6), 6.39 (1H, d, *J*=2 Hz, H-8), 6.89 (1H, d, *J*=8 Hz, H-5*'*), 7.57 (1H, dd, *J*= 2 and 7.5 Hz, H-6′), 8.02 (1H, d, *J*=2 Hz, H-2′), 3.95 (3H, s, OMe), 5.32 (1H, d, *J* = 7.6 Hz, H-100), 3.4-3.7 (4H, m, H-2ʹʹ, H-3ʹʹ, H-4ʹʹ, H-5ʹʹ), 3.82 (1H, dd, *J* = 11.0 and 7.0 Hz, H-6ʹa), 4.21 (1H, dd, *J* = 11.0 and 4.0 Hz, H-6ʹb) [9, 10].

| (A) | (B) |
| --- | --- |
| 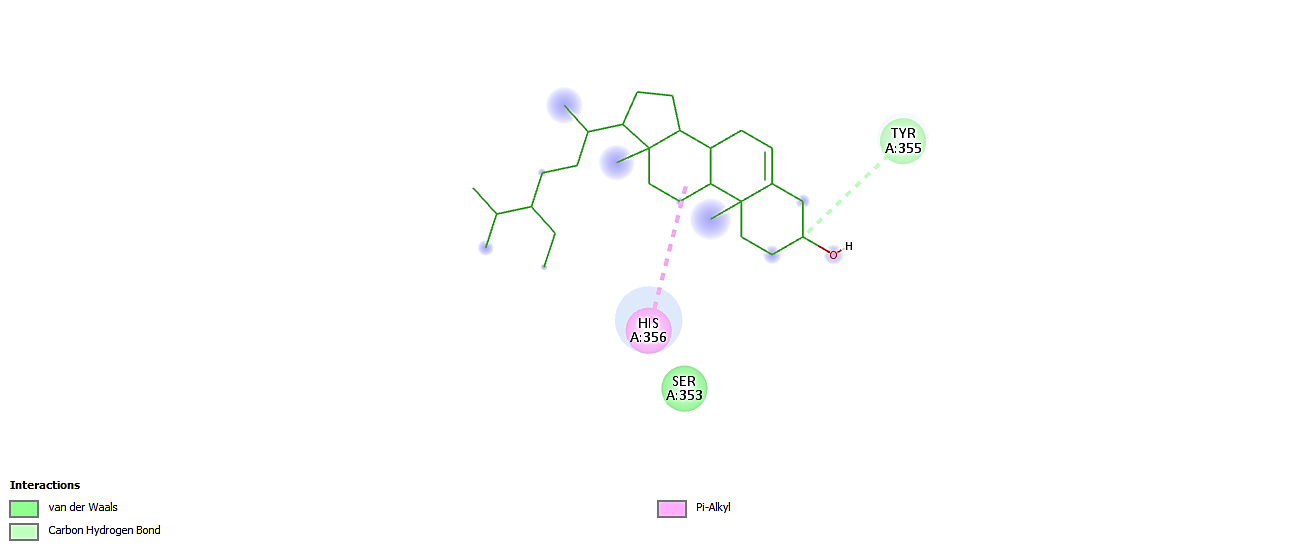 | 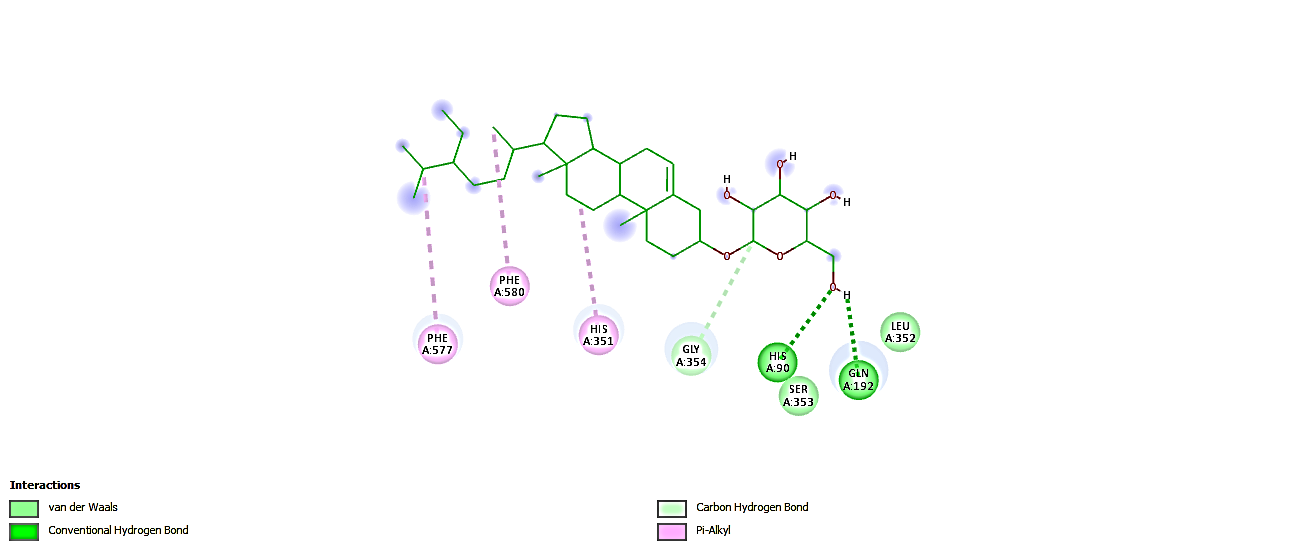 |
| (C) | (D) |
| 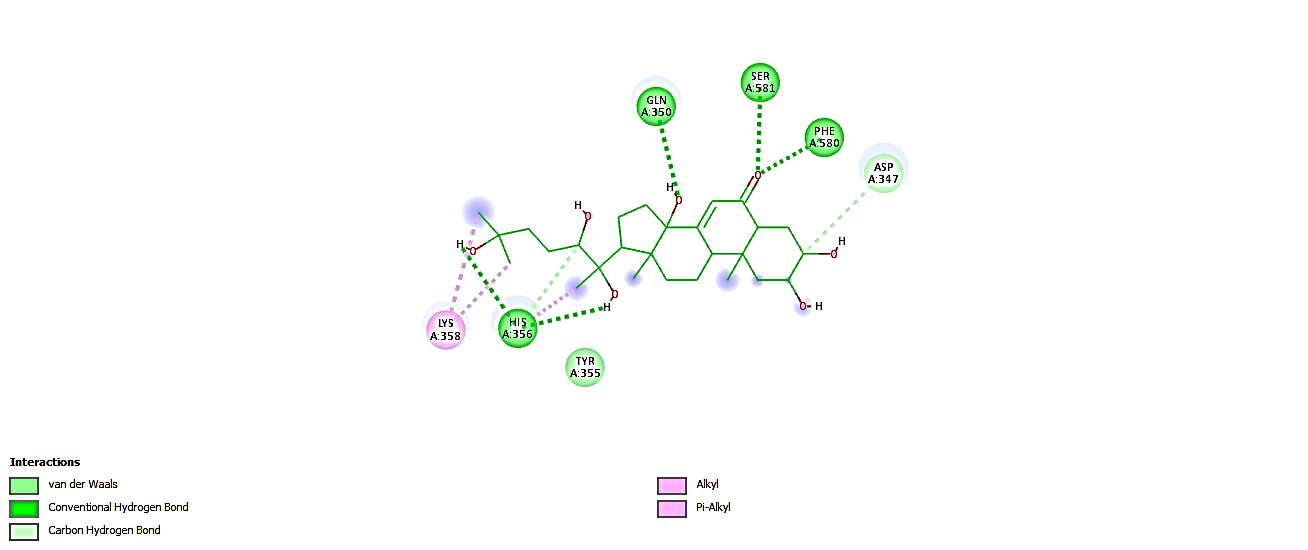 | 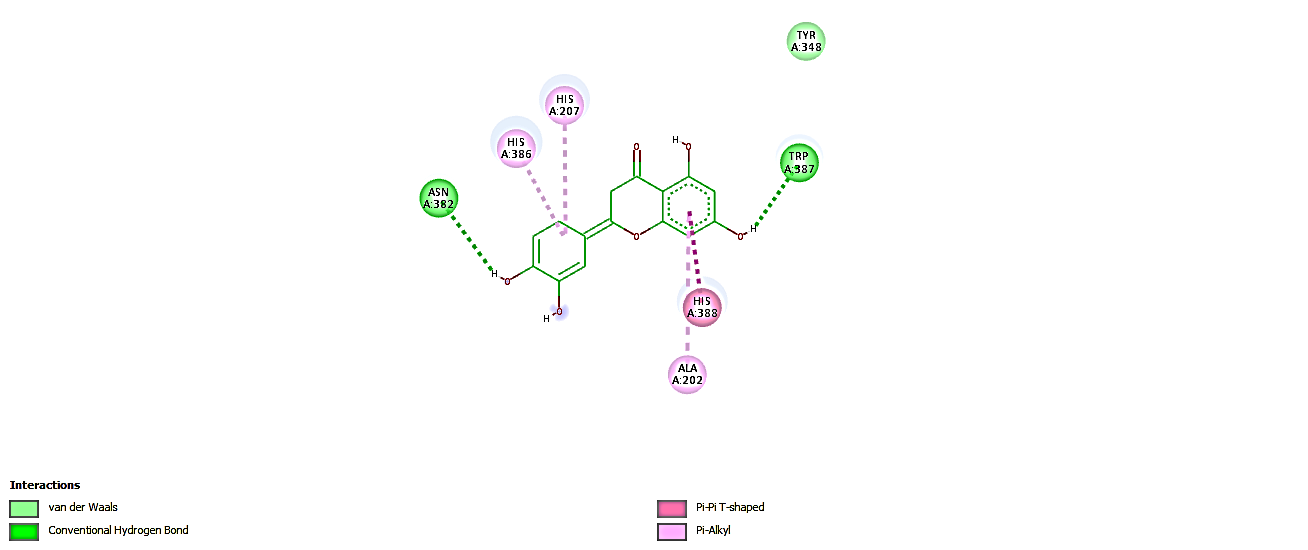 |
| 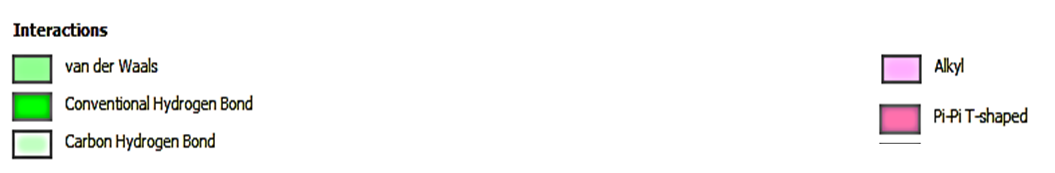 | |
| Figure S1: 2D Docking poses of *β*-sitosterol (A), *β*-sitosterol-3-O-*β*-D-glucopyranoside (B), 20-hydroxy-ocdysone (C), and luteolin (D) in the active site of COX-2. | |

# References:

1. Girault J, Lafont Rd. The complete ^1^H-NMR assignment of ecdysone and 20-hydroxyecdysone. Journal of insect physiology. 1988;34(7):701-6.

2. Thang PT, Dung NA, Giap TH, Oanh VTK, Hang NTM, Huong TT, et al. Preliminary study on the chemical constituents of the leaves of *Macaranga balansae Gagnep*. Vietnam Journal of Chemistry. 2018;56(5):632-6.

3. Elwekeel AH, Amin E, Khairallah A, Moawad AS. *Terminalia arjuna* flowers: Secondary metabolites and antifungal activity. Pharmaceutical Sciences Asia. 2022;49(3):249-56.

4. Ruksilp T. Fatty Acids and an Ester from the Leaves of Millettia utilis Dunn. Naresuan University Journal: Science and Technology (NUJST). 2020;28(3):63-8.

5. Afifi NI, Moawad AS, Hetta MH, Mohammed RM. Phytochemical composition and antioxidant activity of two species related to family Arecaceae. Pharm Sci Asia. 2022;49(1):43-50.

6. Sadikun A, Aminah I, Ismail N, Ibrahim P. Sterols and sterol glycosides from the leaves of *Gynura procumbens*. Natural Product Sciences. 1996;2(1):19-23.

7. Liu W-B, Hu L, Hu Q, Chen N-N, Yang Q-S, Wang F-F. New resveratrol oligomer derivatives from the roots of *Rheum lhasaense*. Molecules. 2013;18(6):7093-102.

8. Li L, Henry GE, Seeram NP. Identification and bioactivities of resveratrol oligomers and flavonoids from *Carex folliculata* seeds. Journal of agricultural and food chemistry. 2009;57(16):7282-7.

9. Güvenalp Z, Demirezer L. Flavonol glycosides from *Asperula arvensis* L. Turkish Journal of Chemistry. 2005;29(2):163-9.

10. Wang Y, Guo T, Li JY, Zhou SZ, Zhao P, Fan MT, editors. Four flavonoid glycosides from the pulps of *Elaeagnus angustifolia* and their antioxidant activities. Advanced Materials Research; 2013: Trans Tech Publ.
